# Genome-wide association analysis of forage quality in maize mature stalk

**DOI:** 10.1186/s12870-016-0919-9

**Published:** 2016-10-21

**Authors:** Hongwu Wang, Kun Li, Xiaojiao Hu, Zhifang Liu, Yujin Wu, Changling Huang

**Affiliations:** Institute of Crop Science, Chinese Academy of Agricultural Sciences, Beijing, 100081 China

**Keywords:** Genome-wide association, Maize, Forage quality, Digestibility

## Abstract

**Background:**

Plant digestibility of silage maize (*Zea mays* L.) has a large influence on nutrition intake for animal feeding. Improving forage quality will enhance the utilization efficiency and feeding value of forage maize. Dissecting the genetic basis of forage quality will improve our understanding of the complex nature of cell wall biosynthesis and degradation, which is also helpful for breeding good quality silage maize.

**Results:**

Acid detergent fiber (ADF), neutral detergent fiber (NDF) and in vitro dry matter digestibility (IVDMD) of stalk were evaluated in a diverse maize population, which is comprised of 368 inbred lines and planted across seven environments. Using a mixed model accounting for population structure and polygenic background effects, a genome-wide association study was conducted to identify single nucleotide polymorphisms (SNPs) significantly associated with forage quality. Scanning 559,285 SNPs across the whole genome, 73, 41 and 82 SNPs were found to be associated with ADF, NDF, and IVDMD, respectively. Each significant SNP explained 4.2 %–6.2 % of the phenotypic variation. Underlying these associated loci, 56 genes were proposed as candidate genes for forage quality.

**Conclusions:**

Of all the candidate genes proposed by GWAS, we only found a *C3H* gene (*ZmC3H2*) that is directly involved in cell wall component biosynthesis. The candidate genes found in this study are mainly involved in signal transduction, stress resistance, and transcriptional regulation of cell wall biosynthetic gene expression. Adding high digestibility maize into the association panel would be helpful for increasing genetic variability and identifying more genes associated with forage quality traits. Cloning and functional validation of these genes would be helpful for understanding the molecular mechanism of the fiber content and digestibility. These findings provide us new insights into cell wall formation and deposition.

**Electronic supplementary material:**

The online version of this article (doi:10.1186/s12870-016-0919-9) contains supplementary material, which is available to authorized users.

## Background

Maize (*Zea mays L*) is not only an important staple crop feeding billions of people but also a main forage resource for animal rearing development. Forage maize is a type of high-energy silage that supplies dry matter, organic matter and cell walls of whole plants for ruminants. The forage feeding value is usually denoted as the utilization ratio of the transformation from forage constituents to energy used by the animal. Improving the feeding value is a major objective in forage maize breeding by increasing forage digestibility, especially cell wall digestibility.

Cell walls protect plants from pests and microbial infection [1, 2] and are involved in stress sensing and signal transduction [3]. In higher plants, cell walls are mainly composed of cellulose, hemicelluloses, pectins, proteins and lignin [4]. The amount and composition of these cell wall components differ among various plant cell types [5]. Variations in cell wall structure and composition have an essential effect on plant digestibility. Among the cell wall components, lignins are important for structure integrity of stalk tissues, and contribute to the mechanical support of plants [6]. However, association and cross linkages between lignins with other cell wall components greatly increase the resistance to degradation or stover fermentation. A balance must be maintained between a robust cell wall architecture and increasing forage digestibility. Thus, quantification of cell wall related traits is necessary for forage quality breeding.

To estimate lignin content of forage plants, the acid detergent lignin/neutral detergent fiber (ADL/NDF) was first proposed by Goering and Van Soest (1970) [7]. NDF mainly consists of cellulose, hemicelluloses and lignins [8], which corresponds to the cell wall content [8, 9]. After hemicelluloses are solubilized with acid detergent treatment. cellulose and lignins are left as residual parts of cell wall,the main part of ADF [10]. Consequently, hemicellulose content can be determined by NDF minus ADF, and cellulose content is assumed to be the difference between ADF and ADL [10, 11]. For digestibility, IVDMD is a routine method to evaluate whole plant enzymatic solubility. And different IVDMD methods were developed by several studies [12]. However, the chemical methods for measuring fiber content and digestibility are complex to perform and costly in breeding programs. Prediction equations based on near infra reflectance spectroscopy (NIRS) was developed and widely used in estimating cell wall content and plant digestibility [10].

Due to the importance of silage maize in livestock farming, breeders began to select maize based on digestibility levels (preferring high digestibility), which resulted in improved cultivars and germplasm [13]. A set of brown midrib mutants with a reddish-brown midrib pigmentation was found to be responsible for reduced lignin concentration, composition [14] together with increased cell wall digestibility [15]. Among these mutants, *bm1*, *bm3* and *bm5* are known to increase enzymatic degradability of maize cell walls [15]. Until now, *bm1*, *bm2*, *bm3*, and *bm4* have been confirmed to encode cinnamyl alcohol dehydrogenase (CAD) [16, 17], methylenetetrahy-drofolate reductase (MTHFR) [18], caffeic acid O-methyltransferase (COMT) [19] and folylpolyglutamate synthase (FPGS) [20], respectively. However, despite the contributions of lignification level to cell wall indigestibility, the correlation between lignin content and forage digestibility varies in different genetic backgrounds [12]. Penning et al. [21], identified several QTL related to lignin abundance, none of which were associated with enzymatic hydrolysis yield. In several recent studies, lignin levels was demonstrated to not correlate cell wall digestibility by enzyme in several species [22–24]. Thus, plant digestibility can not be improved by merely decreasing the lignin content. Many researchers began to study the genetic basis of cell wall-related traits and digestibility directly. Using linkage mapping, a large number of QTL for forage quality and cell wall digestibility were identified with multiple populations in previous studies [6, 8, 25–39]. Subsequently, a meta-analysis of QTL for plant digestibility and cell wall composition in maize was performed [10]. Twenty-six meta-QTL for digestibility traits were detected using a consensus map of 11 experiments. Approximately 42 % of meta-QTL overlapped with QTL for cell wall, which coincided with trait correlations. Furthermore, 356 potential candidate genes for cell wall biosynthesis were mapped onto the consensus map, and 39 % of the candidate genes were located within meta-QTL confidence intervals. These studies proposed numerous potential associated loci and genes for silage quality, which need further investigation and validation.

Recently, genome-wide association studies (GWAS) have played an important role in dissecting complex quantitative traits in plants due to faster analyses, numerous high resolution markers, and abundant genomic and phenotypic variation [40]. Maize has extreme genetic and phenotypic diversity, with more rapid linkage disequilibrium (LD) decay than other species [41]. The rapid development of various genotyping technologies has aided the improved resolution of GWAS with tremendous numbers of markers. In maize, GWAS has become a powerful approach that can be successfully used in dissecting the genetic architecture for many traits, but it has not been performed to dissect traits related to forage quality traits in maize. In this study, with an association panel of 368 diverse inbred lines from around the world, we performed a GWAS analysis to dissect the genetic architecture of forage quality and to identify candidate genes for fiber content and plant digestibility.

## Methods

### Germplasm and field experiments

The association panel used in the present study contains 368 diverse inbred lines (AM368), including resources from the International Maize and Wheat Improvement Center (CIMMYT), China and the USA. Most of the lines from CIMMYT belong to tropical or sub-tropical germplasm sources. Detailed information about AM368 was provided in a previous study [42]. These inbred lines were planted in Hainan and Yunnan in 2010; in Hainan, Henan, and Yunnan in 2011; and in Hainan and Yunnan in 2012. A randomized block design was conducted at all locations, without replication. Each line was planted in a single row (2.5 m in length) of 11 plants at a density of 60,000 plants/ha. Adjacent rows were spaced 0.67 m apart.

### Phenotyping methods

In the present study, when each inbred line reached physiological maturity (i.e. a black layer appeared in kernels), the ears were harvested by hand. After harvest, the second to fifth internodes above the ground of six plants from each inbred line were collected with garden scissors. All samples were immediately enzyme-deactivated at 105°C for 30 mins in a forced air oven and air-dried for 10–14 days. Dried stalk samples were ground with a mill and screened through a mesh size of 0.1 mm. Acid detergent fiber (ADF), neutral detergent fiber (NDF) and *in vitro* dry matter digestibility (IVDMD) were detected by NIRS [43, 44]. Before measurement, stalk samples were dried at 45°C for 48 h to exclude the influence of moisture. Samples were scanned through a near-infrared reflectance spectrophotometer (VECTOR22/N; BURKER Optik, Ettlingen, Germany). ADF, NDF, and IVDMD were determined using NIRS prediction equations developed for maize plants. A modified partial least squares approach implemented in OPUS 6.0 Bruker software was used for fitting the calibration equations [45]. The coefficients of determination for cross-validation (*R* ^*2*^_*CV*_) and external validation (*R* ^*2*^_*Val*_) were 93.6 % and 94.6 % for ADF, 95.3 % and 96.5 % for NDF, and 90.2 % and 91.2 % for IVDMD, respectively.

### Genotyping

The genotyping panel consisted of two sets: the MaizeSNP50 BeadChip, containing 56,110 SNPs, and 1.03 million high quality SNPs detected using RNA sequencing [42]. Included in the genetic analysis were 525,105 high-quality SNPs with minor allele frequency (MAF) above 0.05 [46]. After combining these two sets of genotypes and removing the duplicate SNPs, 559,285 SNPs (MAF ≥ 0.05) were used in the GWAS analysis. The information about genotype data was described in Fu et al. (2013) [46]. The genotype data can be downloaded at http://www.maizego.org/Resources.html.

### Statistical analyses

The GLM procedure was performed in SAS to dissect the variance of the phenotypes in different environments. The model used for the analysis of variance was *y*_*i*_ = *μ* + *e*_*l*_ + *f*_*i*_ + *ε*_*li*_, where *y*_*i*_ is the phenotypic value of the “i”th line, *μ* is the grand mean of the target trait, *e*_*l*_ is the environmental effect of the “l”th environment, *f*_*i*_ represents the genetic effect of the “i”th line, and *ε*_*li*_ is denoted as the residual error. All the effects were considered as random effects. The variance components were used to calculate the broad sense heritability as *H* ^2^ = *σ*_*g*_^2^/( *σ*_*g*_^2^ + *σ*_*ε*_^2^/*e*), where *σ*_*g*_^2^ represents the genetic variance, *σ*_*ε*_^2^ is the residual error variance item, and *e* is the number of environments. The 95 % confidence intervals of the *H* ^2^ were calculated following the method of Knapp et al. [47]. The Pearson correlation coefficients between traits were computed using the PROC CORR procedure in SAS.

To eliminate the environmental effect within multiple environments, we fitted a mixed linear model to calculate the best linear unbiased prediction (BLUP) value for each line: *y*_*i*_ = *μ* + *g*_*i*_ + *e*_*i*_ + *ε*_*i*_. In this equation, *y*_*i*_ represents the phenotype of the “i”th line, *μ* is the grand mean value of the target trait in all environments, ${\mathit{\mathsf{g}}}_i$ represents the genetic effect, *e*_*i*_ is the environmental effect, and *ε*_*i*_ is the random error. The grand mean was fitted as fixed effect, and genotype together with environment were considered as random effects. BLUP estimation was obtained by using the MIXED procedure (PROC MIXED) in SAS9.3 (SAS Institute), which should be denoted as the sum of the grand mean and genetic effect of each line. The BLUP values of each line were used as the phenotypic values for association mapping.

### Genome-wide association analysis

A genome-wide association analysis was performed by accounting for the population structure and kinship matrix [48], which were estimated in a previous study [42]. Using ‘no compression’ and ‘population parameters previously determined’ (P3D) algorithms, the MLM program in TASSEL 4.1.26 [49] was utilized to detect the association between the phenotype and genotype. All the candidate genes were annotated according to the information available in MaizeSequence (http://ensembl.gramene.org/Zea_mays/Info/Index) and in the MaizeGDB database (http://www.maizegdb.org/gbrowse).

### Threshold for GWAS

Background LD between the high-density SNPs used in the present study can be problematic for multiple testing [50]. In addition, the cutoff determined by the Bonferroni tests seemed to be too strict for the present study. A less stringent cutoff of 1 × 10^−4^ was applied for the detection of significant associations. To identify the most robust and stable associations, another round of GWAS was performed with a subsampling procedure [51]. Subsampling was performed 100 times with R software [52], and 80 % of inbred lines were randomly sampled each time. Finally, only the SNPs detected as significant at *P* < 1 × 10^−4^ and identified at least 10 times in 100 subsamples (resample model inclusion probability (RMIP) threshold of 0.1) were denoted as significant associations.

### Linkage disequilibrium and candidate gene selection

The linkage disequilibrium measure (*r* ^2^) with each SNP significantly associated with forage quality traits in a region of 1 Mb were estimated in TASSEL 4.1.26 [49]. We also examined the LD between significant SNPs for each trait. If the LD between adjacent peak SNPs > 0.2, they will be grouped into one unique locus. The genes containing or adjacent to significant SNPs were identified using the maizeB73 reference genome assembly v2 available on the MaizeGDB Genome database (http://gbrowse.maizegdb.org/gb2/gbrowse/maize_v2/). The closest gene of each peak SNP was proposed to be the most likely candidate gene.

## Results

### Phenotypic variability, correlation and heritability

**Fig. 1:**
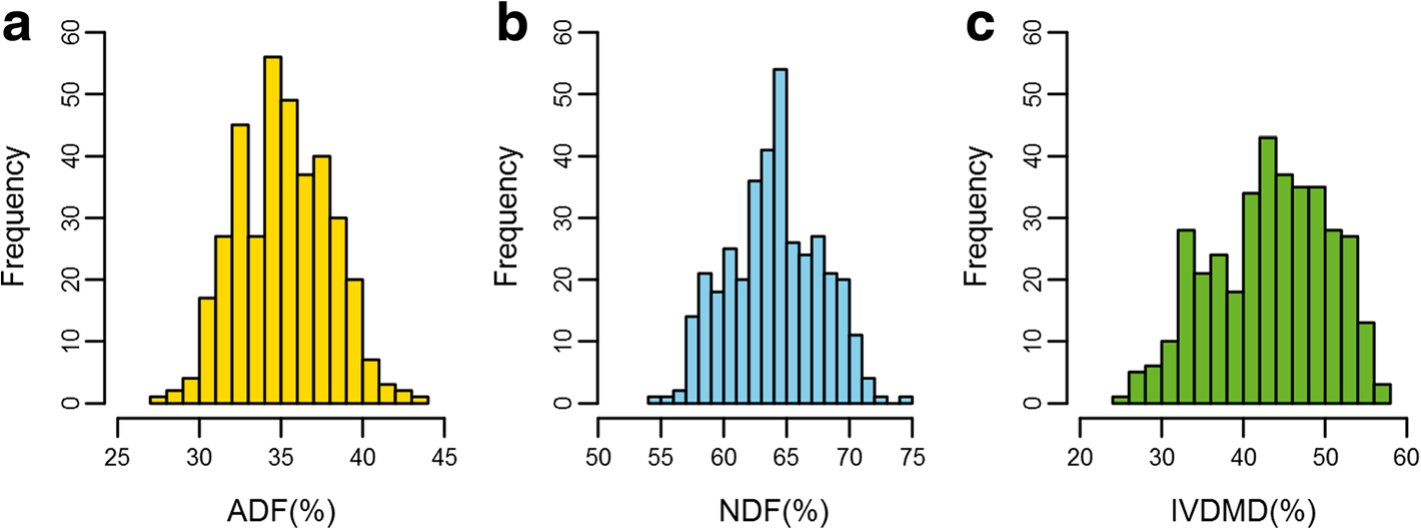
Distribution of forage quality traits in association panel. The frequency distribution of ADF, NDF, and IVDMD in association panel are shown in **a**, **b** and **c**, respectively

**Fig. 2:**
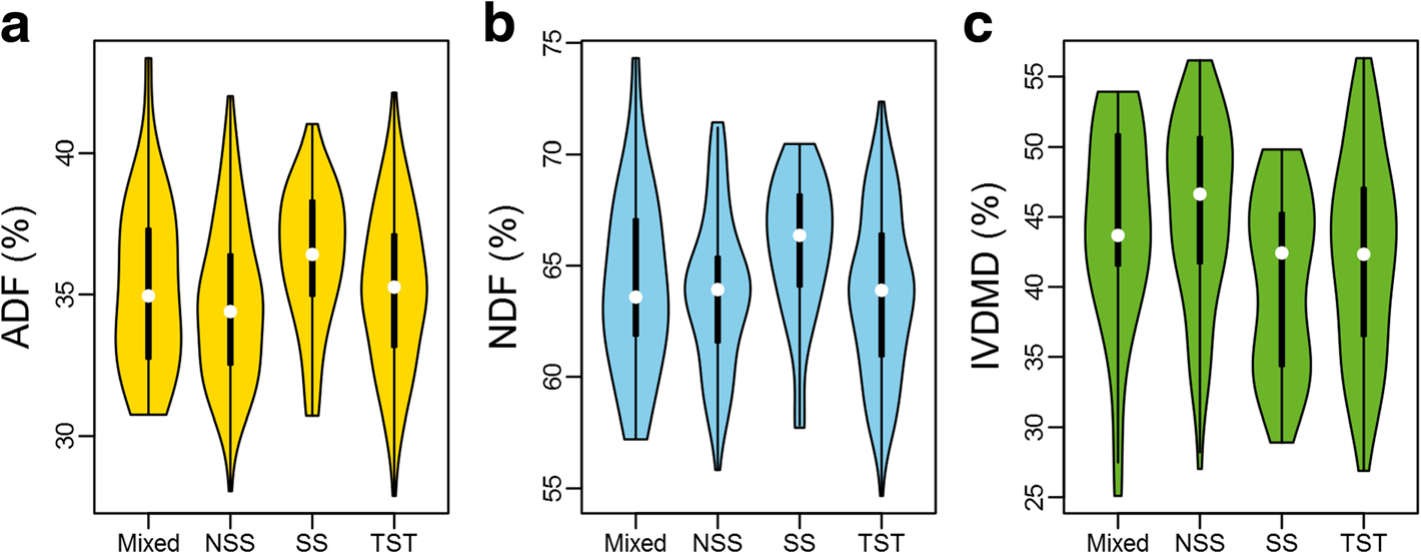
Phenotypic variation of forage quality traits in sub-groups of association panel. The phenotype distribution of ADF, NDF, and IVDMD in each sub-group are shown in **a**, **b** and **c**, respectively. Mixed: Mixed group; SS: Stiff stalk; NSS: Non-stiff stalk; TST: Tropical and sub-tropical

The phenotypic variation of ADF, NDF, and IVDMD in the diverse association panel were assessed using 368 inbred lines in seven environments. The BLUP value of three forage quality traits showed abundant variation, which ranged from 27.9 % to 43.4 % (ADF), 54.7 % to 74.3 % (NDF), and 25.1 % to 56.3 % (IVDMD) with averages of 35.2 ± 2.8 %, 64.1 ± 3.6 %, and 43.2 ± 7.0 %, respectively (Table 1 and Fig. 1). According to a previous study [53], the association panel can be divided into four sub-groups, including Stiff stalk (SS), Tropical–subtropical (TST), Non-stiff stalk (NSS), and Mixed groups. The average ADF and NDF content in the SS group is relatively higher than that of the other three groups. In addition, the dry matter digestibility of the SS group is significantly lower than that of the NSS and MIXED group (Table 1 and Fig. 2). The population structure contributes 3.13 %, 3.09 % and 6.78 % of the variance for ADF, NDF, and IVDMD, respectively.

**Table 1:** Phenotypic variation of forage quality traits in the association panel and subpopulations

	ADF(%)		NDF(%)		IVDMD(%)	
	Range	Mean ± SD	Range	Mean ± SD	Range	Mean ± SD
BLUP	27.9–43.4	35.2 ± 2.8	54.7–74.3	64.1 ± 3.6	25.1–56.3	43.2 ± 7.0
MIXED	30.7–43.4	35.2 ± 3.0	57.2–74.3	64.1 ± 4.0	25.1–53.9	44.4 ± 7.1
NSS	28.0–42.0	34.7 ± 2.8	55.8–71.4	63.8 ± 3.4	27.0–56.2	45.5 ± 6.6
SS	30.7–41.0	36.3 ± 2.4	57.7–70.5	65.8 ± 3.2	28.9–49.8	40.4 ± 6.2
TST	27.9–42.1	35.2 ± 2.8	54.7–72.3	63.8 ± 3.7	26.9–56.3	42.2 ± 7.1

The results of an analysis of variance showed that both genotype and environmental effects significantly affected the forage quality traits (*P* < 0.01). Using the phenotypic value across all environments, the broad sense heritability of each trait was calculated. The heritability estimates of ADF, NDF, and IVDMD were 0.81, 0.79, and 0.85, respectively (Table 2). Significantly positive correlation between ADF and NDF was observed using a correlation analysis (*r* = 0.90, *P* < 0.01), while IVDMD showed a strong negative correlation with ADF and NDF (*r* = −081, *r* = −0.74, *P* < 0.01).

**Table 2:** Analysis of variance, heritability and correlation

Traits	Mean Squares		(*H* ^*2*^)^b^	CI^c^	Correlation		
	E^a^	G^a^			ADF	NDF	IVDMD
ADF	4404.92^**^	68.63^**^	0.81	0.78–0.83	1		
NDF	12449.61^**^	116.44^**^	0.79	0.76–0.82	0.90^**^	1	
IVDMD	16051.09^**^	393.83^**^	0.85	0.83–0.87	−0.81^**^	−0.74^**^	1

^**^Significant at *P* < 0.01

^a^Mean square values for environmental and genotypic factors

^b^Broad-sense heritability

^c^95 % confidence interval of broad-sense heritability

### GWAS of forage quality in mature maize stalk

**Fig. 3:**
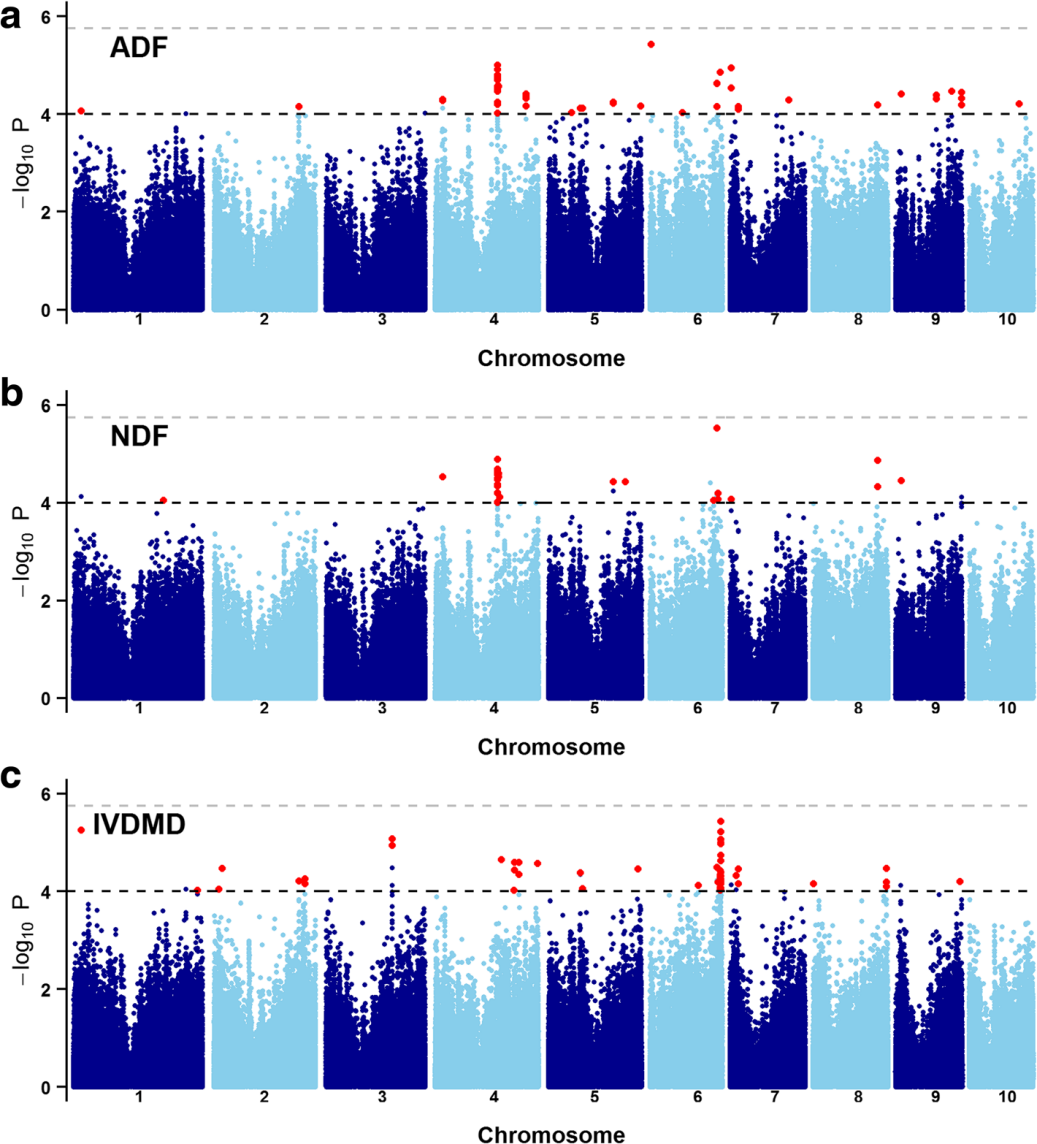
GWAS results for forage quality traits in a maize association panel. Manhattan plots for ADF, NDF, and IVDMD are shown in **a**, **b** and **c**, respectively. *Grey* and *black dashed lines* correspond to the thresholds of Bonferroni correction (*P* < 1.8 × 10^−6^) and *P* < 1 × 10^−4^. *Red dots* indicate significant SNPs (*P* < 1.0 × 10^−4^ and RMIP > 0.1) associated with each cell wall-related trait

Integrated with the population structure and familial relatedness, a mixed linear model was fitted to scan 559,285 SNPs across the whole genome. Quantile-quantile plots (QQ plots) implied that the population structure and familial relatedness were well controlled in the GWAS of each trait (Additional file 1: Figure S1). At a significance level of *P* < 1 × 10^−4^ together with a threshold of RMIP ≥ 0.1, 73, 41 and 82 SNPs were identified as being associated with ADF, NDF, and IVDMD, respectively (Fig. 3, Additional file 1: Figure S1, Table 1). For all three traits, the phenotypic variance explained by each allele (*R* ^*2*^) ranged from 4.2 -6.2 %. SNPs that were significantly associated with ADF (correspond either to cellulose or lignin), which covered 24 unique significant loci, were distributed on all chromosomes except 3 (Fig. 3a and Additional file 2). In addition, 14 loci were found to be significantly associated with NDF (correspond to hemicellulose, cellulose, and lignin) (Fig. 3b), which were located on chromosomes 1, 4, 5, 6, 7, 8 and 9. The maximum number of significant loci for ADF and NDF were both observed on chromosome 4. As shown in Manhattan plots, a set of consecutive SNPs on chromosome 4 were significant for both ADF and NDF (Fig. 3a, Fig. 3b). The SNPs significantly associated with IVDMD were located on all chromosomes except 10. Of the significant SNPs associated with IVDMD, 60 % were located at the distal end of chromosome 6, generating a “hot spot” associated with forage digestibility.

### Candidate gene selection and co-localization between different traits

The MaizeGDB genome database (http://gbrowse.maizegdb.org/gb2/gbrowse/maize_v2/) of the maize B73 reference genome assembly v2 was used to annotate the genes that were near the significant SNPs [54]. After excluding repeated loci caused by co-localization between traits, a total of 56 genes were proposed as candidate genes for the three traits. Among these candidate genes, 10 encode uncharacterized proteins, and the proteins encoded by the remaining genes include transcription factors (GRF, LIM, ARR-B, NAC, bHLH, SBP, and hb3-type transcription factor families), enzymes involved in stress resistance, protein metabolism, signal transduction, cell wall biosynthesis and other biological processes.

**Fig. 4:**
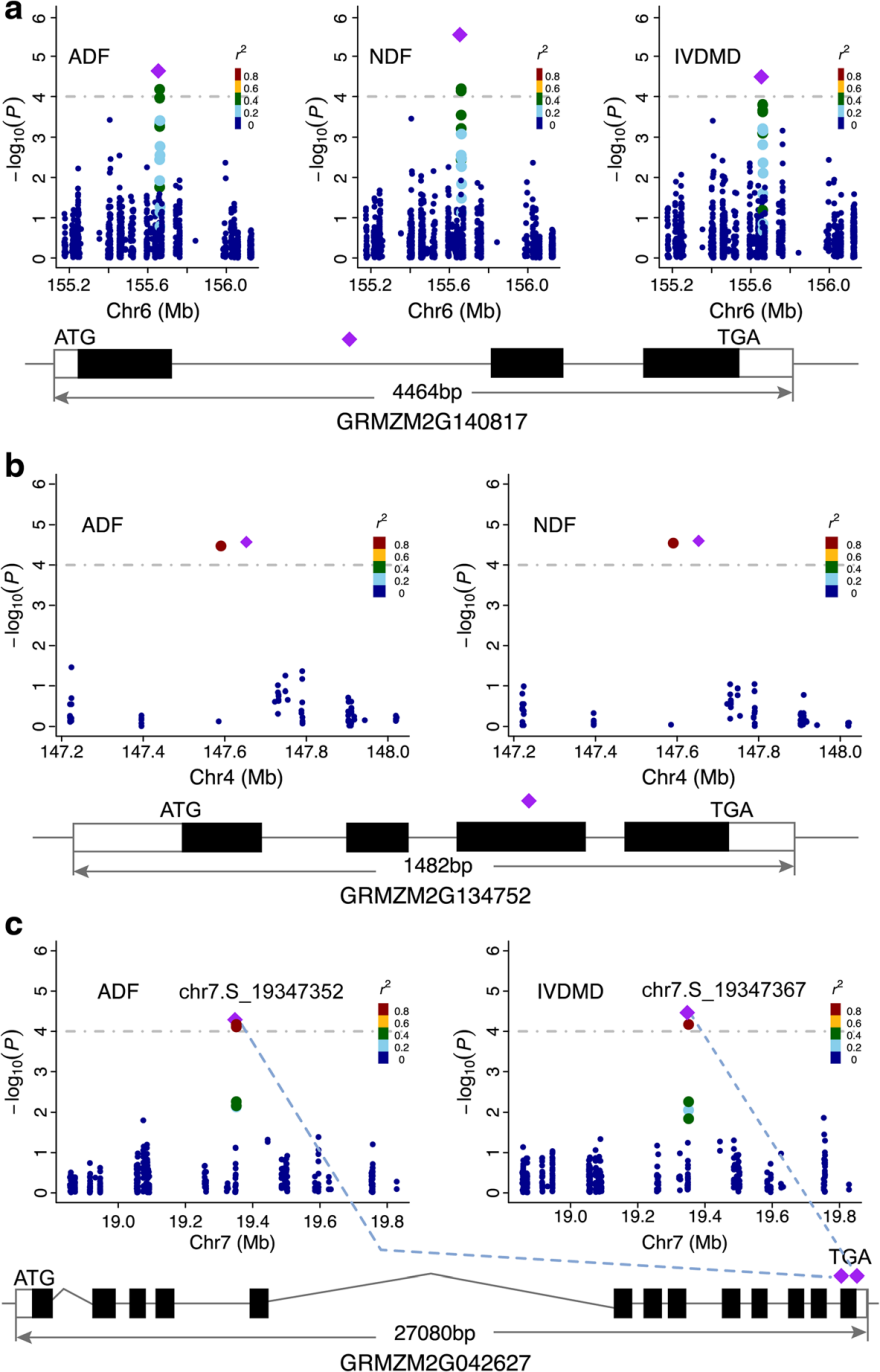
Association and genomic location of known and new loci associated with forage quality traits. (**a**-**c**) Three identified genes were associated with forage quality traits. *GRMZM2G140817* (*ZmC3H2*) (**a**) was associated with ADF, NDF, and IVDMD. *GRMZM2G134752* (**b**) was associated with ADF and NDF. *GRMZM2G042627* (**c**) was associated with ADF and IVDMD, with different leading SNPs. (*Top*) A 0.5-Mb region on each side of the leading SNP, which is denoted by a *purple diamond*. The color of the remaining SNPs reflects the r^2^ values with the most significantly associated SNP. *Dashed horizontal lines* depict the significance threshold (1 × 10^−4^). (Bottom) Gene structure according to the information from the B73 genome sequence in the GRAMENE database (http://ensembl.gramene.org/Zea_mays/Info/Index)

In the GWAS analysis, twelve loci were identified as co-localized associations, which might be associated with two or three traits simultaneously. A strongly associated SNP (chr6.S_155653406) is contained in the gene *GRMZM2G140817*(*ZmC3H2*)*,* which encodes a coumarate-3-hydroxylase that catalyzes hydroxylation reactions of the aromatic ring in the monolignol biosynthesis pathway [55] and significantly associated with the three traits studied herein. This SNP was significantly associated with the three traits (Fig. 3, Additional file 2) and located in the first intron of *ZmC3H2* but did not affect alternative splicing. Relatively moderate LD (R^2^ < 0.6) was observed between adjacent SNPs with the leading SNP (Fig. 4a). On chromosome 4, a significant SNP (PZE-104075114) was found to be associated with both ADF and NDF (Fig. 4b). The corresponding candidate gene containing this SNP encodes a LIM transcription factor (GRMZM2G134752) that regulates the expression level of the genes involved in the lignin biosynthesis pathway [43]. In addition to the two genes mentioned above, a candidate gene for the significant SNP on chromosome 7 at position 19,347,352 (*GRMZM2G042627*) encodes a kinase associated protein phosphatase (KAPP) that is related to the reaction response to pathogen attack and other stresses [56]. Additionally, another leading SNP which is located 15 bp downstream of chr7.S_19347352 was found to be associated with IVDMD (Fig. 4c). These two leading SNPs were both located in the last exon of gene *GRMZM2G042627*, and the level of LD between these two SNPs was high (r^2^ = 0.93, Fig. 4c).

In addition to the three co-localized candidate genes mentioned above, nine more genes were also found to be common candidate genes (Additional file 6). *GRMZM2G331833*, which encodes CLP protease regulatory subunit X, was identified as candidate genes for both NDF and IVDMD. In addition to the LIM transcription factor previously described, the co-localizations between ADF and NDF encode a small ribosomal subunit protein, an aspartic proteinase nepenthesin, an ARR-B-transcription factor, an F-box domain and LRR containing protein and an uncharacterized protein. Furthermore, three more genes were detected as candidate genes for both ADF and IVDMD; the encoded proteins included a GRF transcription factor, a mitogen-activated protein (MAP) kinase, and an uncharacterized protein.

## Discussion

### Phenotypic variation, heritability, and correlation

In the present study, a panel consisting of 368 inbred lines collected from all over the world was used to dissect the genetic architecture of forage quality traits in mature maize stalk. Approximately 1.6-, 1.3- and 2.2-fold variations were observed for ADF, NDF, and IVDMD, respectively. The huge variations in association mapping population suggests that natural germplasm with a broad genetic base could be a potential resource for improving forage quality. The sample size and phenotypic variation in the current study are larger than in the previous studies of associations between lignin pathway genes and forage quality traits [57–60]. According to the population stratification, the association panel was divided into four sub-groups [53]. We found relatively higher fiber content and lower digestibility in the SS group, which contains 45 lines with high stalk strength (data not shown) and is represented by B73, a classic line from the Iowa Stiff Stalk Synthetic (BSSS) maize populations. These results suggest that population structure has an effect on forage quality and confirm the correlation between stalk strength and cell wall-related traits.

The phenotypic variation of the three traits across seven environments were dissected by an analysis of variance. Both genotype and environmental effects were significant for the three traits. In the previous studies about forage quality traits, heritability estimates ranged from 0.51 to 0.92 [30, 31, 36, 38, 39, 57]. In the present study, the heritability estimates for ADF, NDF, and IVDMD in the current study were relative high, 0.79 to 0.85, consistent with previous studies. These results reveal the strong genetic bases of these traits. A significant negative correlation between IVDMD and fiber content (*r* = −0.81 for ADF and −0.74 for NDF) was found, which suggests that high fiber content genotypes will be difficult to digest.

### Genetic architecture of forage quality in mature maize stalk

Population structure can create unexpected LD between loci on separate chromosomes and lead to spurious associations for GWAS [41]. Although population structure contributes only a small proportion of the phenotypic variation in the present study, we still included population structure and familial relatedness matrices into the mixed model of the GWAS. As shown in Quantile-Quantile plots (Additional file 1: Figure S1), false positive associations were well controlled for the GWAS of ADF, NDF, and IVDMD.

GWAS have been a powerful tool for studying the genetic basis of complex traits in maize. Because of the rapid decay of linkage disequilibrium in the association panel, the genome of maize was broken up into small LD blocks. The resolution for identifying an association with ultra-high density SNPs is at the gene level. In the present study, we scanned 559,285 SNPs to identify SNPs associated with ADF, NDF, and IVDMD. With this high-density genotype data, background LD between SNPs may be a problem for multiple testing. The threshold determined by the Bonferroni correction seemed to be too strict for detecting significant associations. To resolve this problem, a suggestive threshold that is lower than the Bonferroni correction with a subsampling procedure were employed in previous studies [61, 62]. In the current study, using *P* < 1 × 10^−4^ and RMIP >0.1 as the significance level, we found 56 unique loci that were significantly associated with forage quality traits. The phenotypic variation explained by each locus ranged from 4.2 %–6.2 %, which revealed that the genetic basis of the three traits in our association panel are mainly controlled by a number of minor effect quantitative trait genes (QTG).

Up to now, hundreds of QTL related to cell wall related traits have been identified across the whole genome of maize [8, 25, 28, 30–32, 34–39, 63, 64]. The previous studies suggest that the genetic architecture of these cell wall-related traits were controlled by a few major QTL and a large number of minor effect QTL, and these cell wall and digestibility QTL cover 77 % and 58 % of the maize genome, respectively [10]. Therefore, it is not surprising that most of the associations detected in the current study were co-localized with previously identified QTL regions. On the other hand, no silage maize was contained in the association panel of this study. The frequency of the favorable allele in silage maize may be reduced in our GWAS analysis. These rare favorable alleles generally explain a large proportion of the phenotypic variance [65]. However, association mapping has limited statistical power for detecting the effects of rare alleles [66], which may lead to a lack of major effect associations for the traits studied herein. In conclusion, combining the results of GWAS with linkage mapping or adding silage germplasm into the association population would be helpful to better understand the genetic architecture of cell wall-related traits.

### Potential candidate genes and underlying pathways

Identifying the genes associated with forage quality is crucial for understanding the molecular mechanism of cell wall biosynthesis. Among the candidate genes found in the present study, *GRMZM2G140817* (*ZmC3H2*), which encodes a *p*-coumarate 3-hydroxylase, was found to be related to ADF, NDF, and IVDMD. This enzyme belongs to the CYP98 cytochrome P450 family and catalyzes the hydroxylation reaction of the aromatic rings of *p*-coumaric acid, then converts the substrate to caffeic acid (Additional file 3: Figure S2). *C3H* provides a watershed between the non-methoxylated *p*-hydroxyphenyl (H) branch and guaiacyl (G)/syringyl (S) branch in the lignin pathway. In *Arabidopsis*, a *C3H*-deficient mutant was reported to be displaying a *reduced epidermal fluorescence* (*ref*) phenotype [67]. This mutant causes a decrease in the lignin content and produces lower amounts of G and S monolignols than the wild type. The downregulation of *C3H* in poplar and alfalfa caused an increase of H lignin units and a reduction of the total lignin content [68–70]. Other than the dicotyledonous species, *C3H* down-regulation was performed in maize in a recent study [71]. *ZmC3H1* knock-down maize plants were generated and led to a moderate increase of *p*-hydroxyphenyl (H) lignin subunits. The authors concluded that *ZmC3H2* might compensate for the reduced level of *ZmC3H1* in these *C3H1* repressed plants. In view of all these results, the two *C3H* genes may together play an important role in the lignin biosynthesis of maize. The functional validation of *C3H* genes should be performed to study the mechanism of how these two genes affect lignin accumulation in maize.

Other than the key enzyme genes participating in the cell wall biosynthesis pathway, the modulation of the transcription level also significantly affects the rate of flux through the secondary cell wall biosynthesis pathway [72]. A number of transcription factors have been found to be involved in the transcriptional network and responsible for regulating the lignin biosynthetic genes [72–75]. The AC-rich element is a common *cis* element contained in the promoter region of most lignin biosynthetic genes. This type of transcription activator is recognized by the DNA-binding domain of R2R3-MYB transcription factors [76–78]. In addition to the MYB transcription factor, a member of the LIM family transcription factor (*Nt*LIM1) has been reported to bind with AC elements and to regulate the expression of lignin biosynthetic genes in tobacco [79, 80]. The suppression of *Nt*LIM1 led to a reduction in the expression level of *PAL*, *4CL*, and *CAD*, which caused a 27 % decrease of lignin content in antisense lines [80]. Furthermore, a group of NAC transcription factors were proven to regulate secondary wall biosynthesis and deposition in various lignification tissues of *Arabidopsis* [81–85]. In the present study, other than one LIM transcription factor (*GRMZM2G134752*) and two NAC transcription factors (*GRMZM2G031200*, *GRMZM5G857701*), five more transcription factor genes (GRF, ARR-B, bHLH, SBP, and hb3-type transcription factor families) were found to be related to fiber content and digestibility (Additional file 2). These results demonstrate the importance of transcriptional regulation in cell wall biosynthesis. Due to the complexity of the process for cell wall biosynthesis and decomposition, the function of five newly found transcription factor genes should be fully validated with a molecular biology analysis.

Plant cells provide not only mechanical strength for the plant stem but also barriers for attacks by insects and diseases [1]. A strong phenotypic correlation between stress resistance and cell wall-related traits was observed during the selection process of breeding programs [86–88]. QTL co-localization also revealed a significant genotypic correlation between cell wall composition and resistance to pests and diseases [1]. Recently, two key enzymes in lignin biosynthesis (HCT and CCoAOMT) were reported to cooperate with the NLR Rp1 protein to regulate the hypersensitive defense response in maize [89]. In the current study, several candidate genes for cell wall-related traits were found to be involved in stress resistance and signal transduction, which includes protein responses to pest, low sulfur, and drought (Additional file 6). A kinase-associated protein phosphatase gene (KAPP) (*GRMZM2G042627*), which was significantly associated with ADF and IVDMD, was also found to be associated with resistance to herbivore attack [62]. These results provide evidence for a connection between cell wall formation and various stress responses.

In previous candidate gene-based association studies, ten lignin biosynthetic genes were investigated to detect associations between polymorphisms and forage quality traits [57–60]. The results showed that polymorphisms within the *PAL*, *ZmC3H1* and *F5H* were associated with forage quality traits. However, these associations were affected by population structure and multiple tests. Only individual polymorphisms in the *4CL1*, *CCoAOMT2* and *ZmPox3* were stably associated with digestibility [57, 59, 60]. In the present study, we did not find any other gene involved in lignin biosynthesis in association with ADF, NDF or IVDMD with the exception of *ZmC3H2*. In addition, the candidate genes identified in the current study mainly included genes encoding transcription factors, protein kinases, and proteins involved in response to stress and other biological processes. Therefore, we conclude that in diverse association panels, only genetic variants of several key enzyme genes in the lignin pathway may play roles in controlling the genetic basis of forage quality. More phenotypic variation of the forage quality may be attributed to the transcriptional regulation of lignin biosynthesis and genes involved in other cell wall biosynthesis pathways.

## Conclusions

In the present study, a genome-wide association study for forage quality traits was conducted with nearly 560 thousand SNPs. There were 24, 14 and 31 loci found to be associated with ADF, NDF, and IVDMD, respectively. Each of these loci contributes a small proportion of the phenotypic variance. Twelve loci were found to be co-localized for the three traits. Excitingly, underlying a significant associated SNP on chromosome 6, a *C3H* gene, which catalyzes a key step in the lignin pathway, was proposed as a candidate gene for all the three forage quality traits. Most of the candidate genes underlying the associated loci were involved in transcriptional regulation of cell wall biosynthesis and the response to stress, suggesting that the transcriptional regulation of cell wall biosynthesis may play a vital role in cell wall formation and deposition. In conclusion, absence of silage maize in the association panel in the present study may be responsible for the lack of cell wall component biosynthesis genes found using GWAS. This study improves our understanding of the genetic basis of forage quality traits and provides insight into potential areas to improve forage digestibility. Future research will focus on cloning and validating the function of the potential candidate genes.

## Additional files

Additional file 1: Quantile-Quantile plots for the GWAS results for ADF, NDF, and IVDMD. The QQ plots for ADF, NDF, and IVDMD are shown in a, b and c, respectively. The horizontal grey solid line and black dashed line correspond to the thresholds of the Bonferroni correction and *P* = 1 × 10^−4^. (TIF 361 kb)
Additional file 2: Summary of leading SNPs and candidate genes significantly associated with forage quality traits. (XLSX 16 kb)
Additional file 3: The phenylpropanoid pathway, adapted from previous studies [55, 89]. 4CL, 4-hydroxycinnamoyl-CoA ligase; C3H, *p*-coumarate 3-hydroxylase; C4H, cinnamate 4-hydroxylase; CAD, cinnamyl-alcohol dehydrogenase; CCoAOMT, caffeoyl-CoA O-methyltransferase; CCR, cinnamoyl-CoA reductase; COMT, caffeic/5-hydroxyferulic acid O-methyltransferase; F5H, ferulate 5-hydroxylase; HCT, hydroxycinnamoyl transferase; PAL, phenylalanine ammonia lyase. (TIF 202 kb)
Additional file 4: Summary of the SNPs significantly associated with ADF. (XLSX 11 kb)
Additional file 5: Summary of the SNPs significantly associated with NDF. (XLSX 10 kb)
Additional file 6: Summary of the SNPs significantly associated with IVDMD. (XLSX 12 kb)

## Abbreviations

ADF: Acid detergent fiber
BLUP: Best linear unbiased prediction
BSSS: The Iowa stiff stalk synthetic
C3H: Coumarate-3-hydroxylase
CAD: Cinnamyl alcohol dehydrogenase
CIMMTY: The international maize and wheat improvement center
COMT: Caffeic acid O-methyltransferase
FPGS: Folylpolyglutamate synthase
GWAS: Genome-wide association studies
IVDMD: In vitro dry matter digestibility
KAPP: Kinase associated protein phosphatase
LD: Linkage disequilibrium
MAF: Minor allele frequency
MTHFR: Methylenetetrahy-drofolate reductase
NDF: Neutral detergent fiber
NIRS: Near-infrared reflectance spectroscopy
NSS: Non-stiff stalk
P3D: Population parameters previously determined
QTG: Quantitative trait genes
QTL: Quantitative trait locus/loci
ref: Reduced epidermal fluorescence
RMIP: Resample model inclusion probability
SNP: Single nucleotide polymorphism
SS: Stiff stalk
TST: Tropical-subtropical
