# Supplementary figures and images for: Genome-wide association analysis of forage quality in maize mature stalk
Source: BMC Plant Biol. 2016 Oct 21;16:227. doi: 10.1186/s12870-016-0919-9 (PMC5073832; doi:10.1186/s12870-016-0919-9)

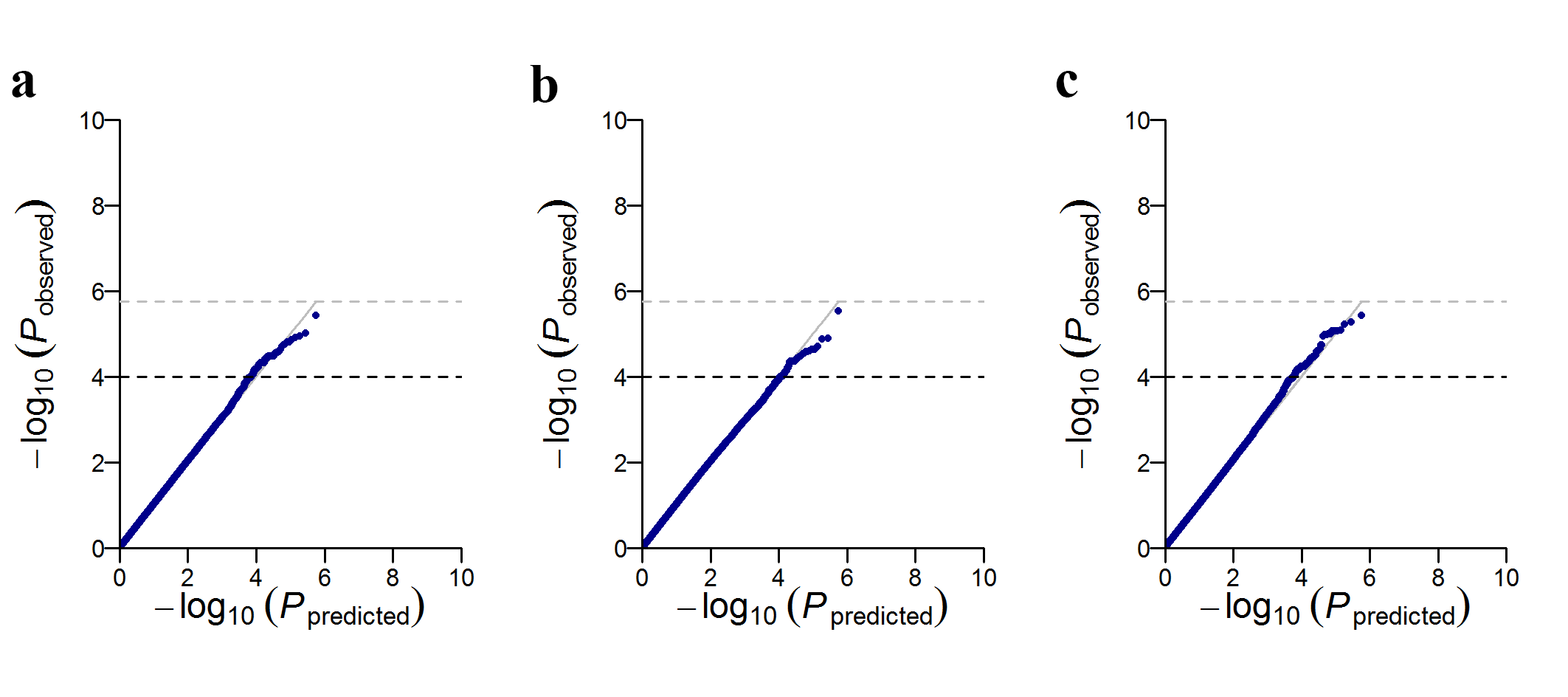

Supplement: Additional file 1: — Quantile-Quantile plots for the GWAS results for ADF, NDF, and IVDMD. The QQ plots for ADF, NDF, and IVDMD are shown in a, b and c, respectively. The horizontal grey solid line and black dashed line correspond to the thresholds of the Bonferroni correction and P = 1 × 10^−4^. (TIF 361 kb) [file 12870_2016_919_MOESM1_ESM.tif]

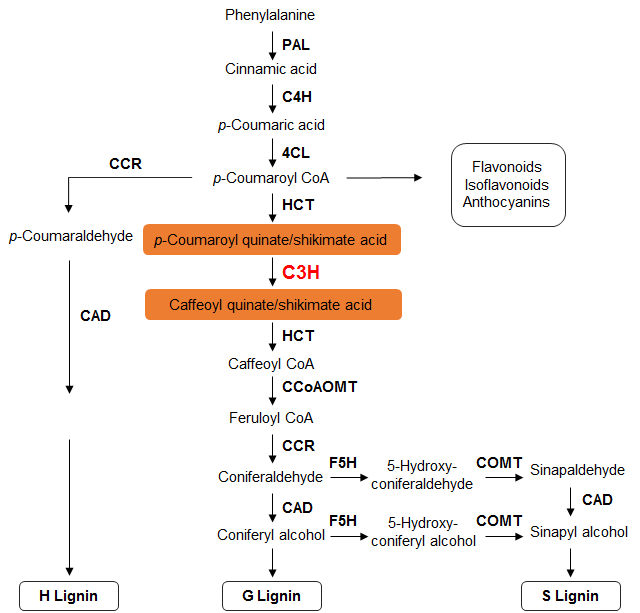

Supplement: Additional file 3: — The phenylpropanoid pathway, adapted from previous studies [55, 89]. 4CL, 4-hydroxycinnamoyl-CoA ligase; C3H, p-coumarate 3-hydroxylase; C4H, cinnamate 4-hydroxylase; CAD, cinnamyl-alcohol dehydrogenase; CCoAOMT, caffeoyl-CoA O-methyltransferase; CCR, cinnamoyl-CoA reductase; COMT, caffeic/5-hydroxyferulic acid O-methyltransferase; F5H, ferulate 5-hydroxylase; HCT, hydroxycinnamoyl transferase; PAL, phenylalanine ammonia lyase. (TIF 202 kb) [file 12870_2016_919_MOESM3_ESM.tif]
